# Identification of the C-terminal region in Amelogenesis Imperfecta causative protein WDR72 required for Golgi localization

**DOI:** 10.1038/s41598-022-08719-7

**Published:** 2022-03-17

**Authors:** Dina Husein, Ahmed Alamoudi, Yoshio Ohyama, Hanna Mochida, Brigitte Ritter, Yoshiyuki Mochida

**Affiliations:** 1 Department of Molecular and Cell Biology, Henry M. Goldman School of Dental Medicine, Boston University, Boston, MA USA; 2 Present Address: Oral Biology Department, Faculty of Dentistry, King Abdulaziz University, Jeddah, Saudi Arabia; 3 Department of Biochemistry, School of Medicine, Boston University, Boston, MA USA

**Keywords:** Cellular imaging, Mechanisms of disease

## Abstract

Amelogenesis Imperfecta (AI) represents a group of hereditary conditions that manifest tooth enamel defects. Several causative mutations in the *WDR72* gene have been identified and patients with *WDR72* mutations have brown (or orange-brown) discolored enamel, rough enamel surface, early loss of enamel after tooth eruption, and severe attrition. Although the molecular function of WDR72 is not yet fully understood, a recent study suggested that WDR72 could be a facilitator of endocytic vesicle trafficking, which appears inconsistent with the previously reported cytoplasmic localization of WDR72. Therefore, the aims of our study were to investigate the tissues and cell lines in which WDR72 was expressed and to further determine the sub-cellular localization of WDR72. The expression of *Wdr72* gene was investigated in mouse tissues and cell lines. Endogenous WDR72 protein was detected in the membranous fraction of ameloblast cell lines in addition to the cytosolic fraction. Sub-cellular localization studies supported our fractionation data, showing WDR72 at the Golgi apparatus, and to a lesser extent, in the cytoplasmic area. In contrast, a WDR72 AI mutant form that lacks its C-terminal region was exclusively detected in the cytoplasm. In addition, our studies identified a putative prenylation/CAAX motif within the last four amino acids of human WDR72 and generated a WDR72 variant, called CS mutant, in which the putative motif was ablated by a point mutation. Interestingly, mutation of the putative CAAX motif impaired WDR72 recruitment to the Golgi. Cell fractionation assays confirmed subcellular distribution of wild-type WDR72 in both cytosolic and membranous fractions, while the WDR72 AI mutant and CS mutant forms were predominantly detected in the cytosolic fraction. Our studies provide new insights into the subcellular localization of WDR72 and demonstrate a critical role for the C-terminal CAAX motif in regulating WDR72 recruitment to the Golgi. In accordance with structural modelling studies that classified WDR72 as a potential vesicle transport protein, our findings suggest a role for WDR72 in vesicular Golgi transport that may be key to understanding the underlying cause of AI.

## Introduction

Amelogenesis Imperfecta (AI) is traditionally defined as congenital enamel formation defects that are hereditary but not accompanied by other morphologic or metabolic defects in the body^1^. AI can be classified according to its mode of inheritance and/or according to the enamel phenotypes clinically and radiographically observed. The abnormality in enamel formation can take place during the secretory stage resulting in hypoplastic AI or in the maturation stage resulting in either hypocalcified or hypomaturation types of AI^2^. Hypoplastic AI may be caused by a mutation in one of the genes responsible for one of the enamel proteins leading to the formation of thin enamel that is fragile and prone to breakage. Hypocalcified and hypomaturation types of AI have normal enamel thickness but the phenotype can be a result of impaired degradation of enamel proteins, which leads to insufficient ions deposition and soft enamel that is easily eliminated^3^.

Mutations in several proteinases including *MMP20* and *KLK4* lead to the improper enamel matrix protein processing and degradation and poor enamel mineralization seen in enamel hypomaturation phenotypes^4^. To date, hypomaturation AI have been associated with mutations in at least four genes that do not encode enamel proteinases, such as pH-sensing G-protein-coupled receptor (GPR68)^5,6^, potassium-dependent sodium/calcium exchanger (SLC24A4)^7,8^, enamel matrix protein phosphorylated by FAM20C (ODAPH)^9,10^ and WDR72^11^.

WDR72 is a member of the WD40 repeat (WDR) domain-containing protein superfamily. WDR proteins typically have recurring units of around 44–60 amino acids that end with tryptophan (W) and aspartic acid (D) and form β sheets that assemble in multi-blade β propellers^12^. WD proteins are often essential subunits of multiprotein complexes involved in a wide range of cellular processes, such as protein–protein interactions, G protein-coupled receptor (GPCR) signaling, DNA damage sensing and repair, the ubiquitin–proteasome system, cell growth and division, epigenetic regulation of gene expression and chromatin organization, and the immune system^13^. The human WDR72 protein is composed of 1102 amino acids with eight WD repeats located in the N terminus part of the protein^11^. WDR72 does not contain a signal peptide, indicating an intracellular localization^14^. The closest homologue to WDR72, WDR7, also known as Rabconnectin-3β in rats, is composed of 1490 amino acids with nine WD repeats. Rabconnectin-3β is involved in the regulation of vesicle mobilization and calcium dependent exocytosis in neurotransmission^15^. The sequence similarities between Rabconnectin-3β and WDR72 suggest that WDR72 may serve a similar function in calcium vesicle turnover^11,16^. A previous study modelling the structure of WDR72 classified WDR72 as a member of the membrane coating family^17^, however, this notion is seemingly inconsistent with the reported cytoplasmic distribution of GFP-tagged WDR72 in cells^14^.

We thus sought to closer examine WDR72 expression in tissues and to use structure–function analysis to determine the subcellular localization of WDR72. Our data demonstrate that WDR72 is a membrane-associated protein that co-localizes with trans-Golgi markers in the perinuclear region. We further identify a putative CAAX motif at the WDR72 C-terminus that is essential for WDR72 recruitment to the Golgi. Together with the proposed role in vesicles transport, our results suggest that the hypomaturation type of AI caused by *WDR72* mutations may be a result of impaired intracellular regulation of proteins critical for correct enamel protein production/formation. Our identification of WDR72 as a Golgi-associated protein opens up important new avenues for understanding the molecular function of WDR72 in amelogenesis.

## Results

### Expression of *Wdr72* in mouse tissues and cell lines

**Figure 1:**
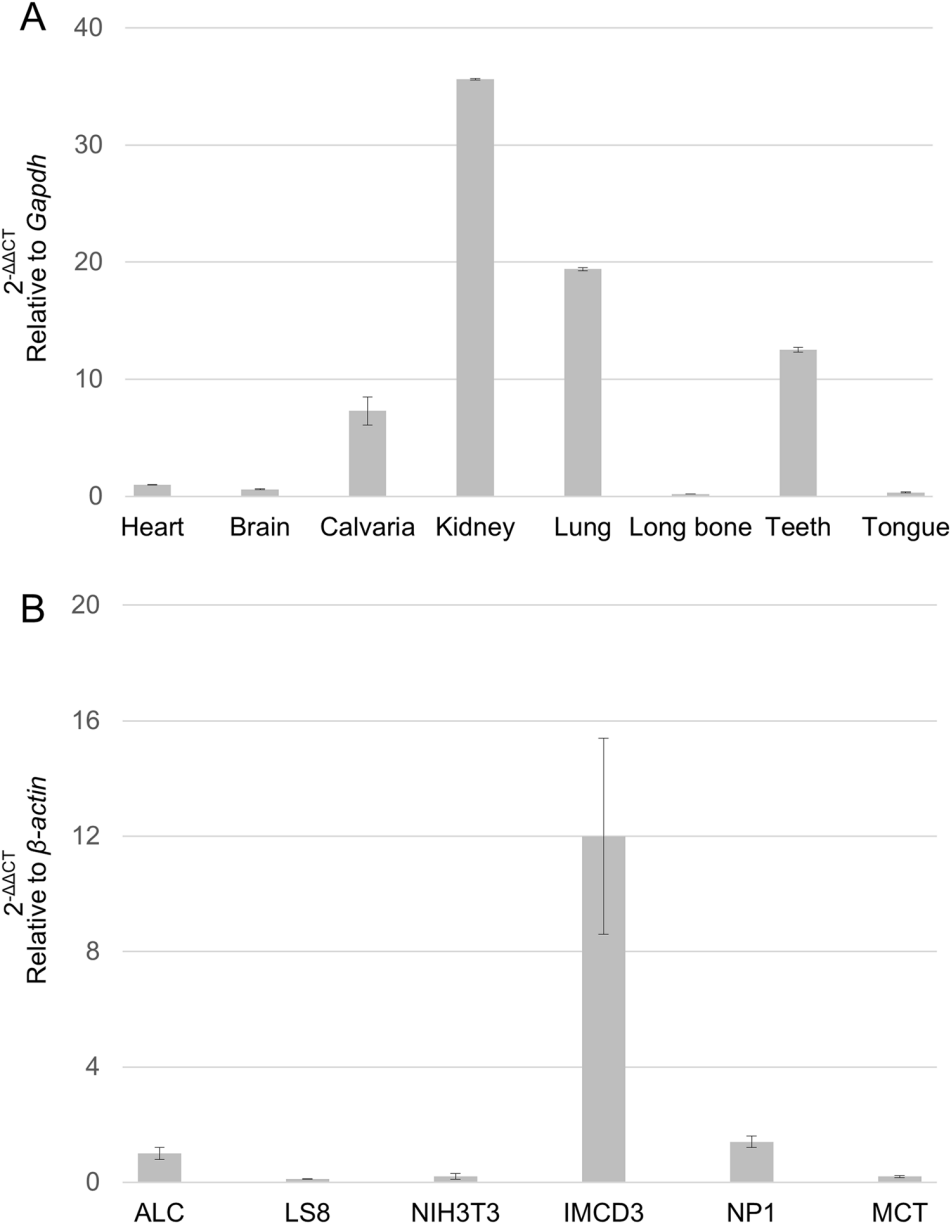
Expression of *Wdr72* in mouse tissues and cell lines. (**A**) The expression of *Wdr72* gene was investigated by quantitative real time-PCR using mouse tissues (heart, brain, calvaria, kidney, lung, long bone, teeth and tongue). The mean fold change in the expression of *Wdr72* was calculated based on the normalization to that of *glyceraldehyde 3-phosphate dehydrogenase* (*Gapdh*) using the value of *Wdr72* in heart as a calibrator. The values are shown as the mean ± SD based on the averages of three independent experiments with triplicates in each. *Wdr72* expression was found to be the highest in kidney followed by lung and teeth, and was found to be the lowest in long bone. (**B**) The expression of *Wdr72* gene was investigated by quantitative real time-PCR using mouse cell lines. Mouse cell lines used included ALC (mouse maturation stage ameloblast-like cells), LS8 (mouse secretory stage ameloblast-like cells), NIH3T3 (mouse embryonic fibroblasts), IMCD3 (mouse kidney inner medullary collecting duct cells), NP1 (murine distal tubular epithelial cells) and MCT (murine proximal tubular epithelial cells). The mean fold change in the expression of *Wdr72* was calculated based on the normalization to that of *β-actin* using the value of *Wdr72* in ALC as a calibrator. The values are shown as the mean ± SD based on the averages of three independent experiments with triplicates in each. The expression of *Wdr72* was found to be the highest in IMCD3 cells, followed by NP1 cells and maturation stage ameloblasts ALC.

To investigate the gene expression of *Wdr72* in various mouse tissues, real-time PCR was performed (Fig. 1A). The results demonstrated that the expression in the kidney was the highest (~ 35 fold of that in heart) among the samples tested. The expression levels in the lung (~ 19 fold of that in the heart), teeth (~ 12 fold of that in the heart) and calvaria (~ sevenfold of that in the heart) were also higher than the other tissues. Lower expression levels were found in brain, tongue and long bone tissues.

We next investigated the gene expression profile of *Wdr72* in six mouse cell lines (Fig. 1B). Since *WDR72* mutation is associated with enamel defects, two ameloblast-like cell lines were chosen, i.e. ALC (mouse maturation-stage ameloblasts) and LS8 (mouse secretory-stage ameloblasts) cells. NIH3T3 (mouse fibroblasts) was also used as a non-odontogenic mouse cell line. As we observed the highest *Wdr72* expression in the kidney (Fig. 1A), IMCD3 (mouse kidney inner medullary collecting duct cells), NP1 (murine distal tubular epithelial cells) and MCT (murine proximal tubular epithelial cells) cells were also analyzed. The expression of *Wdr72* was found to be the highest in IMCD3 cells (~ 12 fold of that in ALC) followed by NP1 cells (~ 1.4 fold of that in ALC). LS8, NIH3T3 and MCT cells showed minimal expressions. The results are consistent with the *Wdr72* tissue expression pattern (Fig. 1A), i.e. cell lines derived from the kidney expressed higher expression levels of *Wdr72*.

### RNA and protein expression of Wdr72 in ALC and LS8 cells

LS8 and ALC cells are two widely-studied ameloblast-like cell lines available in the enamel research community. LS8 cells are less differentiated than ALC cells, indicating LS8 cells are more suitable for studies related to the secretory stage ameloblasts whereas ALC cells are more suitable for maturation stage studies^18^. As AI with *WDR72* mutations presents a hypomaturation phenotype, we tested for WDR72 expression in ALC cells.

**Figure 2:**
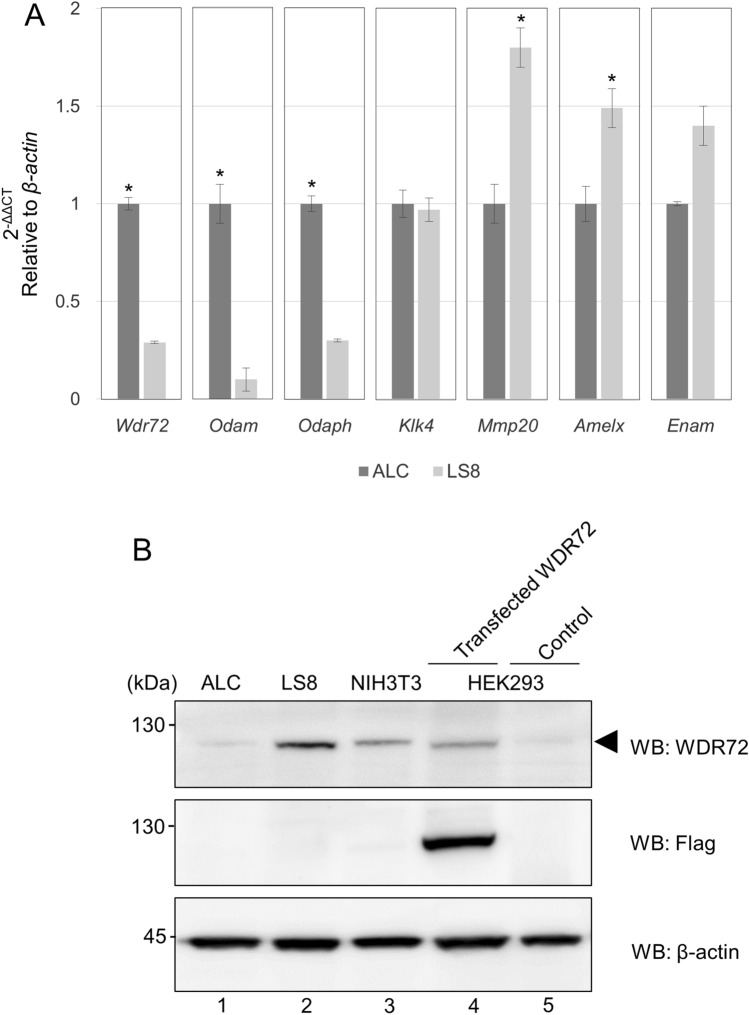
Expression of Wdr72 in ALC and LS8 cells at RNA and protein levels. (**A**) Gene expression of *Wdr72*. The expression levels of amelogenesis gene markers at maturation stage (*Odam*, *Odaph* and *Klk4*) and those at secretory stage (*Mmp20*, *Amelx* and *Enam*) were measured, normalized to that of *β-actin* and compared between ALC and LS8 cells. The normalized value in ALC cells in each gene expression was used as a calibrator. The values are shown as the mean ± SD based on the averages of three independent experiments with triplicates in each. The values in each marker were compared between ALC and LS8 and statistically analyzed by student T-test. An asterisk represents a statistically significant difference between the values of ALC and LS8 in each marker (*p* < 0.01). (**B**) Protein expression of WDR72. Western blot analysis (WB) was performed using ALC, LS8 and NIH3T3 cell extracts to investigate the expression of WDR72 protein by anti-WDR72 antibody. The HEK293 cells were transiently transfected with Flag-tagged human WDR72 wild-type expression vector or an empty vector, and the lysates were used as a positive control (Lane 4, Transfected WDR72) and a negative control for transfection (Lane 5, Control). Western blot analysis by anti-Flag antibody was also performed to confirm the presence of Flag-WDR72 protein. The expression of β-actin was shown as a loading control. The experiment was repeated three times and representative images are shown.

We first investigated the RNA expression levels of amelogenesis gene markers including *Wdr72* in LS8 and ALC cells. Real-time PCR analysis showed that the expression levels of *Wdr72*, *Odam* (*Odontogenic, ameloblast associated*) and *Odaph* (*Odontogenesis associated phosphoprotein*) were significantly higher in ALC cells than those in LS8 cells (Fig. 2A). The expression levels of *Mmp20* and *Amelx* were significantly higher in LS8 cells than those in ALC cells (Fig. 2A), supporting the notion that LS8 cells express secretory stage amelogenesis gene markers. The expression level of *Enam* appeared to be slightly higher in LS8 than in ALC cells, although there was no statistical difference. The expression levels of *Klk4* were unchanged between ALC and LS8 cells. Since *Odam*^19^ and *Odaph*^9^ genes are maturation stage amelogenesis markers, our data indicated that *Wdr72* also belongs to this group. The expression levels of the maturation stage amelogenesis markers including *Wdr72*, *Odam*, *Odaph* and *Klk4* in ALC cells normalized to *β-actin* were also compared, and the data showed that the *Wdr72* demonstrated the least expression level as compared to *Odam*, *Odaph* and *Klk4* in ALC cells (Supplementary Fig. S1).

We next investigated the protein expression level of WDR72 by Western blot analysis using cell lysates extracted from ALC and LS8 cells (Fig. 2B). NIH3T3 fibroblast cells, a non-odontogenic cell line, were used for a control. The cell lysates from HEK293 cells transfected with Flag-tagged human WDR72 were used as a positive control, while those transfected with the empty vector were used as a negative control. Western blot analysis of cells expressing Flag-tagged human WDR72 with anti-WDR72 antibody detected a signal at the molecular weight of around 120 kDa, the predicted size of WDR72 (Fig. 2B, upper panel, lane 4, indicated by an arrowhead). Likewise, Western blot analysis using anti-Flag antibody also detected a 120 kDa band in the Flag-WDR72 transfected cells but not in the empty vector control cell (Fig. 2B, middle panel, lanes 4 and 5), demonstrating that the anti-WDR72 antibody recognizes WDR72. The weak immunoreactivity of the anti-WDR72 antibody with HEK293 cell lysates transfected with the empty vector indicated low levels of endogenous WDR72 in HEK293 cells (Fig. 2B, upper panel, lane 5, Control). In contrast, higher levels of endogenous WDR72 protein were detected in LS8 cells (Fig. 2B, upper panel, lane 2) and to a lesser extent in NIH3T3 cells (Fig. 2B, upper panel, lane 3) and ALC cells (Fig. 2B, upper panel, lane 1).

Our expression data at RNA and protein levels appeared conflicting as *Wdr72* gene was highly expressed in maturation-stage ameloblasts, ALC cells, while WDR72 protein expression was higher in secretory-stage ameloblasts, LS8 than in ALC cells.

### Expression of WDR72 in the cytosolic and membranous fractions of ameloblasts

**Figure 3:**
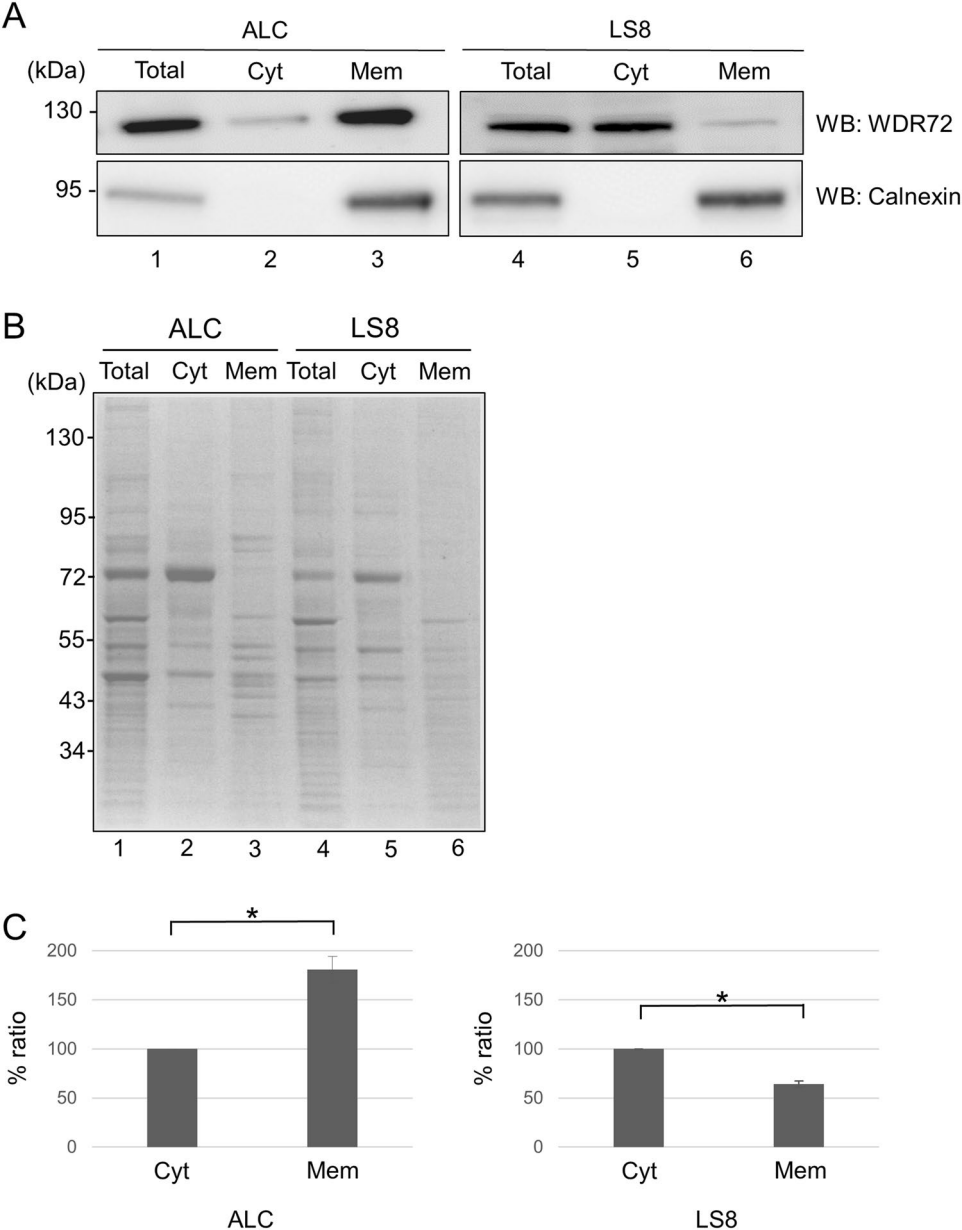
Expression of WDR72 at the cytosolic and membranous fractions in ameloblasts. (**A**) ALC and LS8 cell lysates were fractionated into total cell homogenate (Total), cytosolic fraction (Cyt) and membranous fraction (Mem). Western blot analysis was performed and the expression of WDR72 using these fractions was investigated in both ALC and LS8 cells (upper panels). The successful separation of the membranous fraction from the cytosolic fraction was verified by Western blot analysis with anti-Calnexin antibody (lower panels). The experiment was repeated three times and representative images are shown. (**B**) The equal quantity of extracted protein extracts from each fraction in both ALC and LS8 cells were resolved by 4–12% SDS-PAGE and subjected to Coomassie Brilliant Blue staining. (**C**) The band intensities of cytosolic and membranous fractions immunoreactive to anti-WDR72 antibody in ALC and LS8 cells were measured. The % ratio of the intensities from three different experiments was calculated and the mean values with ± SD are shown. The asterisks show that there was a statistically significant difference between WDR72 protein expression in the cytosolic and membranous fractions (p < 0.01).

The results of our previous experiments showed higher gene expression of *Wdr72*, but lower protein expression in ALC cells as compared to LS8 (Fig. 2). Ectopic expression of WDR72, fused to a 26 kDa GFP tag, revealed s cytoplasmic expression pattern^14^. We, therefore, sought to determine whether endogenous WDR72 protein also localizes in the cytosol of the ameloblast-like cell lines. Both ALC and LS8 cell lysates were fractioned into a total cell homogenate (Total), a cytosolic fraction (Cyt) and a membranous fraction (Mem). Western blot analysis with anti-WDR72 antibody detected a band at the expected molecular weight (~ 120 kDa) in ALC cells in all three fractions (Fig. 3A, upper left panel, lanes 1–3). An immunoreactive band was also detected in LS8 cells in all three fractions (Fig. 3A, upper right panel, lanes 4–6). Western blot analysis using anti-Calnexin antibody detected Calnexin in the membranous fraction (Fig. 3A, lower panels, lanes 3 and 6) and the total cell homogenate (Fig. 3A, lower panels, lanes 1 and 4), but not in the cytosolic fraction (Fig. 3A, lower panels, lanes 2 and 5), demonstrating a successful cell fractionation. Equal quantities of protein samples from each fraction were also prepared, applied to 4–12% SDS-PAGE, and the gel was stained with Coomassie Brilliant Blue R-250 (CBB), confirming the presence of protein in each fraction (Fig. 3B). WDR72 was readily detectable in the membranous fraction (Fig. 3A, upper left panel, lane 3) and, to a lesser extent, in the cytosolic fraction (Fig. 3A, upper left panel, lane 2) of the ALC cells. On the other hand, the expression of WDR72 protein was higher in the cytosolic fraction (Fig. 3A, upper right panel, lane 5) and, to a lesser extent, in the membranous fraction (Fig. 3A, upper right panel, lane 6) of the LS8 cells.

Quantification of WDR72 levels in the different fractions from three independent experiments confirmed that in ALC cells, WDR72 levels in the membranous fraction were significantly higher than in the cytosolic fraction (Fig. 3C, left, ALC). On the other hand, in LS8 cells, WDR72 levels in the membranous fraction was significantly lower than in the cytosolic fraction (Fig. 3C, right, LS8).

### WDR72 localizes at the Golgi apparatus

Our data showed that the endogenous WDR72 was detected in the membranous fraction (Fig. 3), however, WDR72 does not have signal peptide sequence, and thus is not a transmembrane protein^14^. Furthermore, structural modeling suggested that WDR72 resembles vesicle coat proteins^17^. Therefore, we next investigated the sub-cellular localization of WDR72. During the course of our anti-WDR72 antibody characterization, the antibody used in this study clearly showed the approximately ~ 120 kDa band as compared to other anti-WDR72 antibodies tested, which corresponds to the predicted molecular weight of the endogenous WDR72 protein (Figs. 2B and 3A). However, as shown in the supplementary materials, the uncropped Western blot data (for Fig. 2B) showed not only the WDR72 band but also additional bands. Therefore, unfortunately the immunofluorescence against endogenous WDR72 in ALC and LS8 cells was not possible as any signal detected would also contain signals from other proteins, and we selected COS-7 cells, which have a well-spread and flat morphology that allows for high spatial resolution in microscopy imaging, with Flag-WDR72 transient transfection.

**Figure 4:**
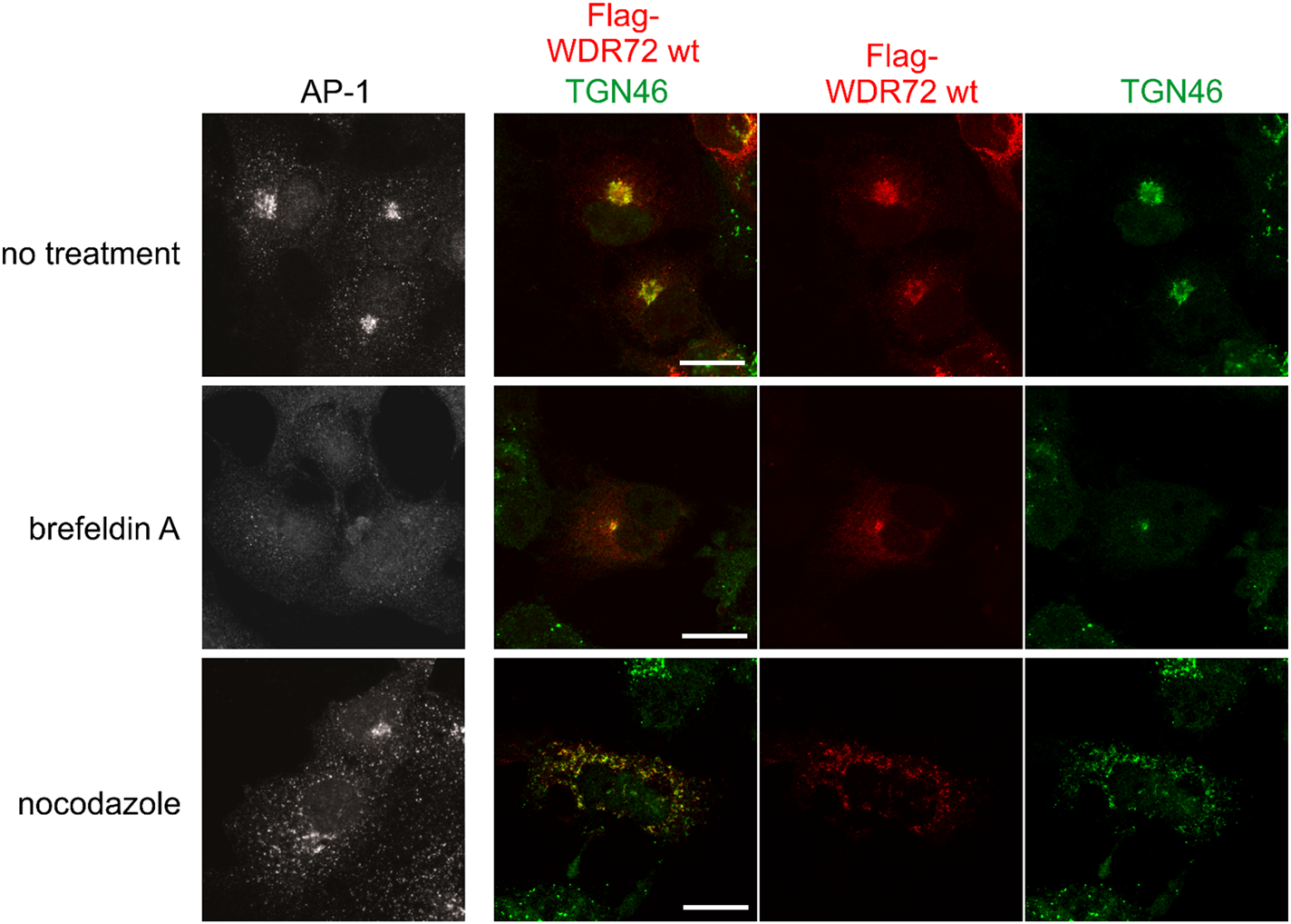
WDR72 localizes at Golgi. COS-7 cells were transiently transfected with Flag-tagged WDR72 wild-type (wt) and the immunolocalization of WDR72 was investigated using anti-Flag antibody together with the endogenous Golgi marker TGN46. Brefeldin A and nocodazole were used to change the morphology of the Golgi whether these drugs affect WDR72 localization. The transfected cells with Flag-WDR72 wt showed the punctate patterns with perinuclear accumulation, co-immunostained with anti-TGN46 antibody, demonstrating that WDR72 localizes at the Golgi. Scale bar represents 25 μm.

Flag-tagged WDR72 showed perinuclear accumulation in addition to cytoplasmic immunoreactivity (Fig. 4, upper panels, right middle, indicated by Flag-WDR72 wt). We then investigated whether WDR72 is co-localized with TGN46, an integral membrane marker for the trans-Golgi network (Fig. 4, upper panels, right, indicated by TGN46). The results demonstrated that the localization of WDR72 perinuclear accumulation is overlapped with that of TGN46 (Fig. 4, upper panels, left middle, indicated by Flag-WDR72 wt TGN46), indicating that WDR72 is recruited to the Golgi apparatus.

To further verify the localization of WDR72 presumably at Golgi, WDR72 transfected cells were treated with brefeldin A (Fig. 4, middle panels indicated by brefeldin A), which inactivates the small GTPase Arf1^20^, thereby blocking AP-1 recruitment to the membrane and blocking clathrin vesicle formation at the Golgi. Our brefeldin A treatment was successful as AP-1 recruitment at the Golgi (Fig. 4, upper panel, left, no treatment) was impaired (Fig. 4, middle panel, left, brefeldin A). Upon brefeldin A treatment, WDR72 WT was re-distributed in the perinuclear region (Fig. 4, middle panels, right middle indicated by Flag-WDR72 wt), similar to the changes seen for TGN46 (Fig. 4, middle panels, right indicated by TGN46) and maintaining the partial co-localization of both proteins (Fig. 4, middle panels, left middle indicated by Flag-WDR72 wt TGN46), suggesting that WDR72 is associated with the TGN.

Nocodazole treatment leads to the formation of Golgi mini stacks, which remain functional but are dispersed in the cytoplasm instead of concentrating in the perinuclear region^21^. Relocalization of the trans-Golgi clathrin adaptor AP-1 demonstrated that the treatment successfully dispersed the Golgi into mini stacks (Fig. 4, left panels, upper-no treatment vs lower-nocodazole). Notably, both TGN46 and WDR72 redistributed in response to nocodazole while maintaining their co-localization (Fig. 4, lower panels, right indicated by TGN46 vs right middle indicated by Flag-WDR72 wt). These data demonstrate that WDR72 is associated with the Golgi membranes and confirm the subcellular cell fractionation results showing WDR72 in the membranous fractions by Western blot analysis.

### Amelogenesis Imperfecta mutant W978X form abrogates WDR72 recruitment to the Golgi

**Figure 5:**
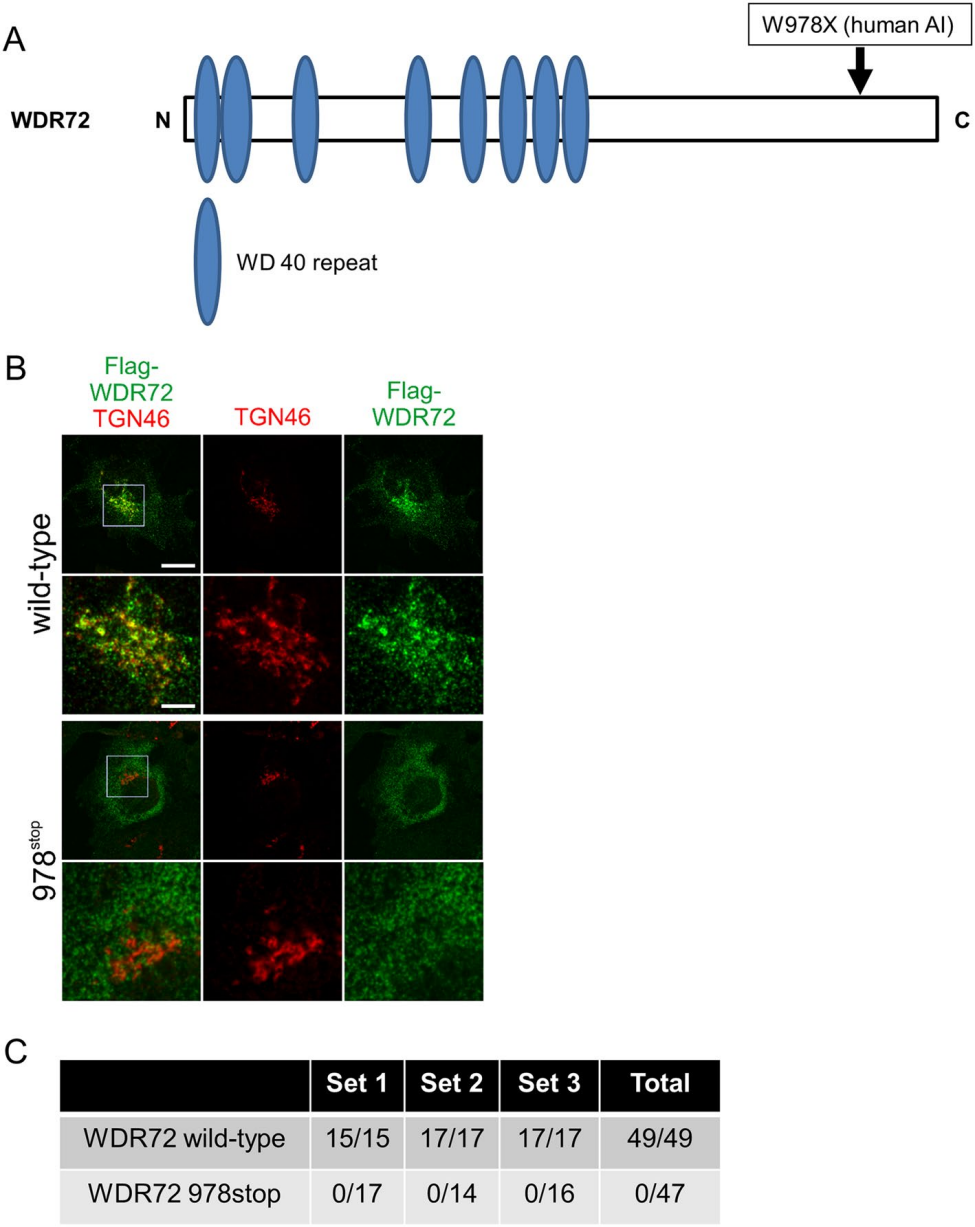
Wild-type WDR72, but not the Amelogenesis Imperfecta (AI) mutant WDR72 form, enriches in the Golgi area. (**A**) Schematic presentation of WDR72 protein with WD 40 repeat motif. The location of W978X mutation is indicated by an arrow for the reference. (**B**) Co-immunofluorescence analysis in COS-7 cells of Flag-WDR72 wild-type and the Amelogenesis Imperfecta (AI) mutant W978stop in comparison with the TGN46 was performed. The lower panels for each WDR72 (wild-type or 978^stop^) show a higher magnification of the area boxed in the merged panel, and of the respective corresponding area in TGN46 and Flag-WDR72 panels shown at a lower magnification. Scale bar represents 25 μm and 5 μm in the low and high magnification images, respectively. (**C**) Quantification of wild-type and AI mutant WDR72 forms localization to the Golgi. To quantitatively analyze the localization of wild-type and AI WDR72 forms, cells were randomly selected per each experiment and these co-localizations were evaluated. Immunofluorescence imaging of co-staining for the endogenously expressed trans-Golgi marker TGN46 was used to score recruitment of the WDR72 forms to the Golgi. The number of cells with a pool of WDR72 at the Golgi and the total number of cells analyzed for each condition are shown for three independent repeats of the experiment (Set 1, Set 2 and Set 3).

WD40 repeat (WDR) domain proteins utilize WD 40 repeat motifs for protein–protein interaction^22^. The WDR region of WDR72 shares 58% sequence similarity with its closest homolog, WDR7, the human counterpart of Rabconnectin-3β^15^, suggesting that the less conserved C-terminal region may serve a different molecular function. To better understand the role of C-terminal region in WDR72, we decided to analyze the subcellular localization of the AI-linked form of WDR72, the c.2934G>A mutation that results in the nonsense mutation at tryptophan residue at position 978 (p.W978X)^11^. As this AI mutation is located outside the WDR-region, the mutant protein retains all WD repeat motifs (Fig. 5A).

While the full-length Flag-tagged WDR72 protein was enriched in the perinuclear region and co-localized with TGN46 (Fig. 5B, left panels, top and upper middle, indicated by Flag-WDR72 TGN46, wild-type), the truncated W978X mutant showed a cytoplasmic distribution with no accumulation at the TGN (Fig. 5B, left panels, lower middle and bottom, indicated by Flag-WDR72 TGN46, 978^stop^).

To quantitatively analyze the localization of Flag-WDR72 wild-type and W978X proteins with co-staining for TGN46, cells were randomly selected per each experiment and these co-localizations were evaluated. As shown in Fig. 5C, the results clearly showed that W978X AI mutant form was not at all localized at the Golgi, but appeared to be dispersed in the cytoplasmic areas.

### The C-terminal CAAX motif is critical for WDR72 Golgi localization

**Figure 6:**
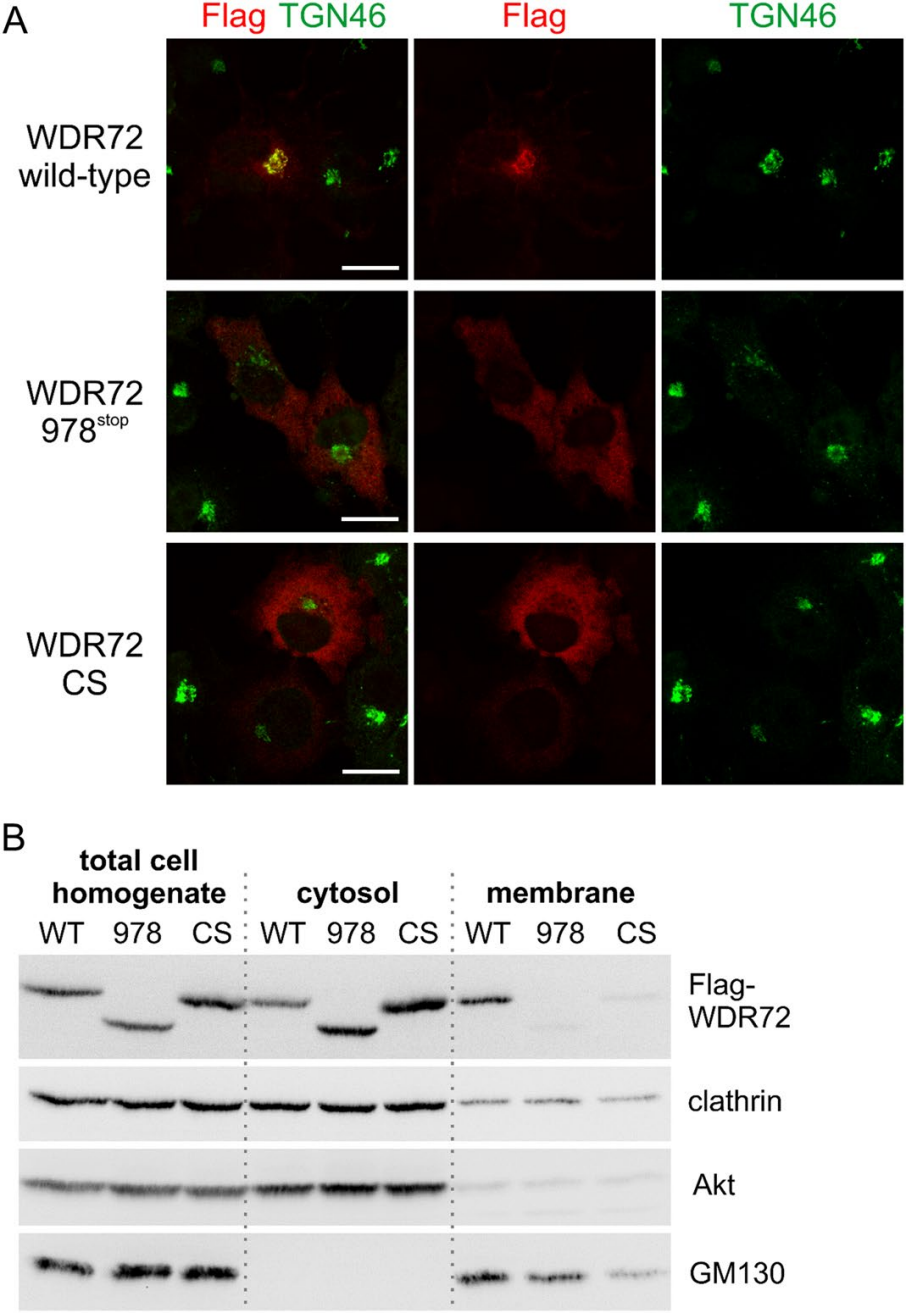
The C-terminal CAAX motif is required for WDR72 localization in the Golgi area. (**A**) The immunolocalization of Flag-tagged WDR72 wild-type, AI WDR72 mutant form (978^stop^), and CAAX mutant form (CS) was investigated using anti-Flag antibody together with the endogenous Golgi marker TGN46. The immunolocalization of CS form was found to be in the cytoplasmic region similar to that of AI WDR72 mutant form. Scale bar represents 25 μm. (**B**) The CAAX mutant, WDR72 CS form, is predominantly detected in the soluble cytosolic fraction. Cell fractionation assay was performed and the expression levels of WDR72 wild-type (WT), AI mutant form (978) and CAAX mutant form (CS) were evaluated. WDR72 WT was detected in both the cytosolic and membranous fractions with slight enrichment in the membranous fraction, while 978 and CS were very predominantly soluble. Anti-clathrin, anti-Akt and anti-GM130 antibodies were used to verify the successful separation of cytosolic and membranous fractions.

The WDR72 W978X mutant demonstrates that the C-terminal region after amino acid 978 is required for the Golgi localization. During inspection of the protein sequence, we noted the presence of the CKVS sequence, a putative prenylation CAAX motif (C: cysteine, A: aliphatic amino acid), at the very C-terminus (aa 1099–1102) of human WDR72. To test whether the putative CAAX motif is important for WDR72 localization, we generated a Flag-tagged CAAX mutant form (CS), in which the cysteine (C) at a position 1099 was mutated to serine (S). Immunofluorescence microscopy of transiently transfected COS-7 cells showed that as previously shown, wild-type WDR72 was enriched at the Golgi apparatus and co-localized with TGN46 (Fig. 6A, upper panels), while the AI-linked mutant form WDR72 W978X showed a diffuse localization in the cytoplasm and no enrichment in the perinuclear region (Fig. 6A, middle panels), confirming our previous results. Notably, the CAAX mutant WDR72 CS also showed a cytoplasmic distribution with no enrichment at the TGN46-marked TGN (Fig. 6A, lower panels).

Subcellular fractionation of HEK 293 cells transfected with the three Flag-tagged WDR72 constructs showed the enrichment of the integral cis-Golgi membrane protein, GM130 in the membranous fraction and the cytosolic Akt kinase in the cytosolic and membranous fractions, respectively, with both proteins present in the total cell lysates (Fig. 6B). Clathrin, a multimeric protein complex formed by heavy and light chains, is the hallmark component of clathrin-coated vesicles and known to cycle between a cytosolic pool and a membrane-bound pool at the TGN, endosomes, and the plasma membrane. Western blotting using an antibody directed against clathrin heavy chain to detect the complex confirmed the presence of endogenous clathrin in both the cytosolic and membranous fractions (Fig. 6B, clathrin). Wild-type WDR72 was present in the total cell lysate, the cytosolic and the membranous fractions (Fig. 6B, Flag-WDR72, WT). The W978X and CS mutant forms of WDR72 were also detected in the total cell lysate at levels comparable to the wild-type proteins (Fig. 6B, Flag-WDR72, 978 and CS). However, both mutant forms were only present in the cytosolic fraction and absent from the membranous fraction (Fig. 6B), confirming that the WDR72 C-terminus and the CAAX motif are essential for WDR72 recruitment to trans-Golgi membranes.

## Discussion

Previously, the gene expression pattern of *Wdr72* in tissues was only described in a non-quantitative manner using the expression sequence tag database where it was found to be the highest in bladder, kidney and mouth^14^. Our results showed the highest expression of *Wdr72* in the kidney and kidney cells such as IMCD3 and NP1. The IMCD3 cells were originally derived from the inner medullary collecting duct of the SV40-transgenic mouse, retaining many characteristics of this particular nephron segment^23^. NP1 cells were generated from freshly isolated tubuli from a C57BL/6J mouse, representing a cell type of the distal tubular kidney epithelial cells^24^. Our findings in WDR72 expression suggest a molecular connection between ectoderm-derived ameloblasts and kidney epithelial cells, supporting the recent reports that WDR72 mutations are associated with distal renal tubular acidosis and Amelogenesis Imperfecta^25–28^.

Amelogenesis is mainly composed of two stages; the secretory and maturation stages. Specific markers are shown to be expressed at a certain time of the life of ameloblasts indicating the stage of amelogenesis. Secretory ameloblasts transition into maturation ameloblasts to perform stage-specific functions with stage-specific gene expressions^29^. The known maturation stage markers of ameloblasts include Odam^19,30^, Odaph^9,10^ and Klk4^31^, while secretory stage markers are Mmp20^32^, Amelogenin^33^ and Enamelin^34^. There are two major ameloblast-like cell lines frequently used and characterized by the enamel research community. One is LS8, where cells from mouse enamel organ epithelia were extracted. The expression of amelogenin was confirmed when the LS8 cell line was created^35^. Another is ALC (ameloblast-like cells), which were prepared from the tooth germs of newly born mice and after multiple isolations, a group of cells were maintained, then ALC cells were established. Moreover, these cells were capable of producing calcified nodules^36^. As a result, LS8 cells tended to express sets of enamel genes and gene products at the secretory stage of amelogenesis, whereas ALC cells expressed those at the maturation stage of amelogenesis^18^. Our results in Fig. 2A were consistent with the previous report that ALC cells carry on a behavior that seems to be more of the maturation stage while LS8 cells tend to behave more toward the secretory stage^18^. Furthermore, our results demonstrated that the gene expression of *Wdr72* was significantly higher in ALC cells than that in LS8 cells (Fig. 2A) indicating that this gene expression is highly upregulated during the maturation stage of amelogenesis. The relative expression level of *Wdr72* in ALC was not higher than any other known maturation amelogenesis markers including *Odam*, *Odaph* and *Klk4* (Supplementary Fig. S1). Although the expression level is moderate, it would be helpful in future studies using the *Wdr72* gene as a maturation marker.

When the expression level of WDR72 protein was compared, the results showed some inconsistency with the gene expression data, i.e. the level of *Wdr72* gene expression was higher in ALC cells than that in LS8 cells (Fig. 1B), while that of WDR72 protein was higher in LS8 than that in ALC (Fig. 2B). It was, therefore, decided to perform cell fractionation to compare the level of WDR72 protein at the cytosolic and membranous fractions between ALC and LS8 cells (Fig. 3). Our results demonstrated that the level of WDR72 protein in the membranous fraction of the ALC cells was significantly higher than that in the cytosolic fraction of the ALC cells, while the results in LS8 cells showed the opposite. Although the differences between RNA and protein levels could possibly depend on RNA stability, protein stability, RNA translation rates, etc., it may be interesting to investigate in the future study that ALC cells may contain a higher level of potential CAAX-motif signal enzyme(s) that could post-translationally modify the WDR72 protein than LS8 cells. For example, Ras GTPases have been well studied for their membrane trafficking via a C-terminal CAAX-motif^37^. The initial post-translational modification is catalyzed by prenyltransferases to attach a substrate-specific lipid to the CAAX cysteine, and the prenyl-CAAX motif is then recognized by a specific proteinase, resulting in the processing of AAX residues. Finally, the prenyl-cysteine is recognized by a prenylcysteine carboxyl methyltransferase that methylesterifies the α carboxyl group^38^. These sequential post-translational modification steps are highly protein sequence-dependent molecular events^39^.

**Figure 7:**
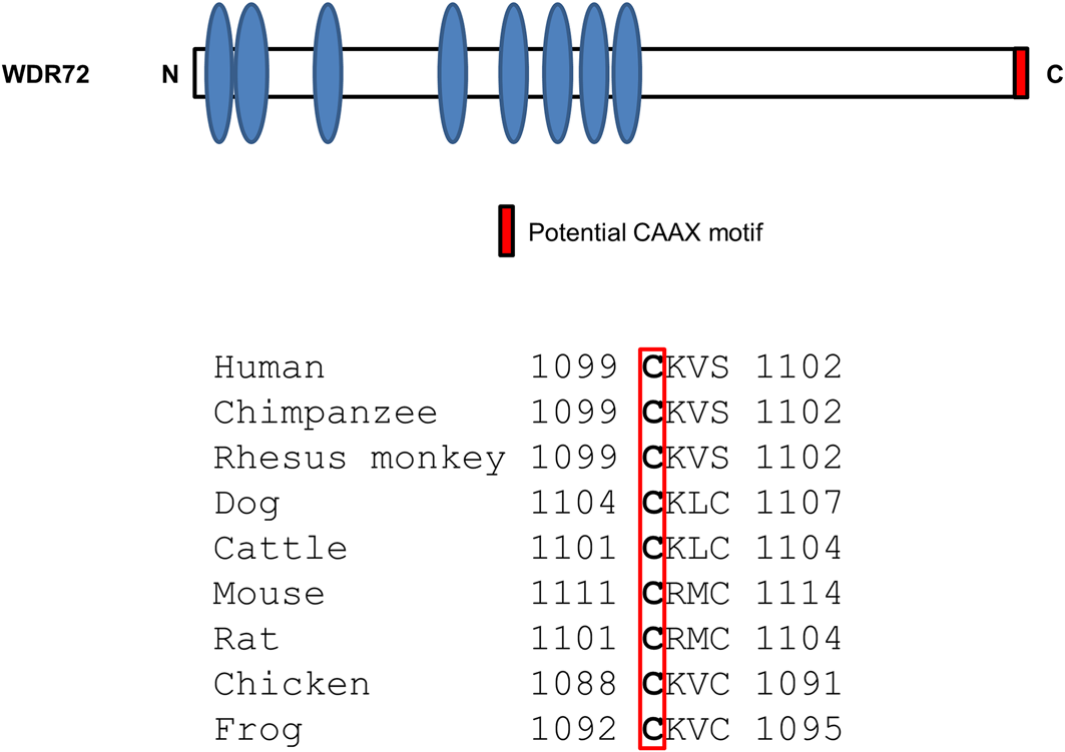
Potential CAAX motif is evolutionarily conserved among species. Schematic presentation of WDR72 protein domain structure (upper illustration). Each domain is symbolized such as WD 40 repeat and potential CAAX motif. The multiple sequence alignment of the last four amino acids containing potential CAAX motif (lower illustration). Protein accession number of WDR72 protein for each organism is as follows: human; NP_877435.3, chimpanzee; XP_003314726.1, rhesus monkey; XP_001088410.1, dog; XP_005638485.1, cattle; XP_002690944.1, mouse; NP_001028672.2, rat; NP_001129191.1, chicken; XP_004943846.1, frog; XP_002936387.2.

It has been so far suggested the molecular function of WDR72 to be involved in calcium transport and/or enamel matrix protein removal^17,40^ mainly by the studies using *Wdr72* knockout mice, however, these outcomes could be indirect consequences of the aberrant signaling pathways. Proteins localizing at the plasma membrane and in particular to clathrin-coated pits at the plasma membrane have a very distinctive distribution. Based on the distribution pattern seen in our immunofluorescence results (Figs. 4 and 5B), there was no noticeable amount showing that pattern. Although this doesn't mean that there is none at all but WDR72 certainly does not concentrate at the plasma membrane, our current data did not support the involvement of WDR72 in endocytosis. The data obtained from this study may provide insights into the potential molecular function of WDR72 in amelogenesis. (1) The immunolocalization of WDR72 protein was accumulated at the Golgi. This is inconsistent with the initial report that the WDR72 localization appeared to be detected only within the cytoplasmic area^14^. This discrepancy could likely be due to a large N-terminal GFP tag (~ 26 kDa) previously used^14^, which might interfere with the proper tertiary structure, in comparison to a smaller N-terminal Flag-tag (~ 1 kDa) used in this study. (2) Although we cannot rule out that the non-sense-mediated mRNA decay may be the major cause for Amelogenesis Imperfecta pathogenesis with *WDR72* mutations, the current study proposes an important first step towards the mechanistic insights as to how the prematurely truncated WDR72 AI mutant proteins, lacking the C-terminal CAAX motif, lead to a loss of WDR72 molecular function. It is of note that there is only one missense mutation of WDR72 AI cases reported^26^, which likely has a different molecular mechanism resulting in enamel defects. (3) As WDR72 does not contain a signal peptide sequence, the WDR72 protein synthesis must occur in the cytoplasm and the protein then likely undergoes the post-translational modification, by which WDR72 protein could translocate to the membranous region. It is well known that H- and N-Ras, c-Able kinase, Src kinase and the α subunits of most heterodimeric G proteins are covalently modified with lipids^41^. In particular, Ras GTPases are targeted from the cytosol to the Golgi and also the plasma membrane^42^ by a series of enzymes modifying the C-terminus of its CAAX and other motifs as mentioned above. One of these modifications, prenylation, is catalyzed by a molecular complex of farnesyltransferase subunits, if the residue in the X position is either alanine, serine, cysteine, methionine or glutamine^38,43^. In the case of WDR72, the cysteine residue of the CAAX motif is evolutionally 100% conserved among all species and the residue in the X position is either serine or cysteine (Fig. 7). It is of our particular future interest to further investigate the role of post-translationally modified WDR72 protein and its molecular function in both healthy enamel tissues and Amelogenesis Imperfecta.

## Materials and methods

### Ethics statement

The use of animals and all animal procedures in this study were approved by the Institutional Animal Care and Use Committee (IACUC) at Boston University Medical Campus (approved protocol number: AN-15233), and all efforts were made to minimize suffering animals. This study was performed in accordance with the NIH Guide for the Care and Use of Laboratory Animals as well as the ARRIVE guidelines.

### Cell culture

All cell cultures in this study were maintained in Dulbecco’s Modified Eagle’s Medium (Life Technologies) supplemented with 10% fetal bovine serum (FBS; Millipore Sigma), 100 units/ml penicillin and 100 µg/ml streptomycin in a 5% CO2 atmosphere at 37 °C. The media were refreshed twice a week. Human embryonic kidney (HEK) 293 (ATCC; CRL-11268), ALC (maturation-stage ameloblast like cells)^36^, LS8 (secretory-stage ameloblast like cells)^35^, NIH3T3 (mouse fibroblasts, ATCC; CRL-1658), IMCD3 (mouse kidney inner medullary collecting duct cells), NP1 (murine distal tubular epithelial cells), MCT (murine proximal tubular epithelial cells) and African green monkey kidney COS-7 (ATCC; CRL-1651) cells were used in this study.

### Reagents and antibodies

X-tremeGENE 9 DNA transfection reagent was obtained from Roche Life Science (used for Fig. 2B). JetPRIME was obtained from Polyplus Transfection (used for immunofluorescence imaging analyses). Lipofectamine 2000 was obtained from Thermo Fisher Scientific (used for fractionation with transfection studies). Brefeldin A and nocodazole were obtained from Millipore Sigma (B6542 and M1404, respectively).

The antibodies used in this study were as follows; rabbit polyclonal anti-WDR72 (HPA057410, Millipore Sigma), anti-Flag (clone M2, F1804, Millipore Sigma), rabbit monoclonal anti-β-actin (#4970, Cell Signaling), rabbit polyclonal anti-Calnexin (ab10286, Abcam), anti-AP-1/γ-adaptin (clone 88, 610385, BD Biosciences), anti-TGN46 (ab50595, Abcam), anti-clathrin heavy chain (clone 23, 610499, BD Biosciences), anti-Akt (#9272, Cell Signaling Technology), and anti-GM130 (clone 35, 610823, BD Biosciences) antibodies.

### RNA extraction and quantitative real-time PCR

To investigate the gene expression of *Wdr72* in various tissues, total RNA was extracted with TRIzol reagent (Life Technologies) using mouse tissues, i.e. heart, brain, calvaria, kidney, lung, long bone, teeth and tongue (C57BL/6, 5-week-old, male) and mouse cell lines, i.e. ALC, LS8, NIH3T3, IMCD3, NP1 and MCT cells. Two µg of the total RNA extract was used for reverse transcription (RT) using the Omniscript RT Kit (Qiagen). Real-time PCR was performed in triplicate using the specific TaqMan primers-probe for mouse *Wdr72* (Applied Biosystems, ABI assay number; Mm006260010_m1), rodent *glyceraldehyde-3-phosphate dehydrogenase* (*Gapdh*; 04308313, for Fig. 1) or mouse *β-actin* (Mm01205647_g1 for Fig. 2A and Supplementary Fig. S1), and the expression levels were analyzed by the ABI 7300 real-time PCR system. For *Wdr72* expression in mouse tissues, the mean fold changes in the expression of *Wdr72* relative to that of *Gapdh* were calculated using the values obtained from the heart cDNA as a calibrator by means of 2^-ΔΔCT^ method as we previously reported^44^. For *Wdr72* expression in mouse cell lines, the mean fold changes in the expression of *Wdr72* relative to that of *β-actin* were calculated using the values obtained from the ALC cDNA as a calibrator by means of 2^−ΔΔCT^ method. To investigate the expression levels of amelogenesis gene markers in ALC and LS8, real time-PCR was performed as described above using the values of the ALC cDNA as a calibrator in each gene expression analysis. The expression levels of the amelogenesis gene markers including *Wdr72*, *Odam*, *Odaph* and *Klk4* using the maturation stage ALC cells were measured by real-time PCR analysis, and the values based on the normalization to that of *β-actin* was compared by means of 2^−ΔCT^ method.

The following markers were used: *Wdr72*, *Odam* (Odontogenic, ameloblast associated, Mm02581573_m1), *Odaph* (Odontogenesis associated phosphoprotein, Mm02020633_s1), *Klk4* (Kallikrein related peptidase 4, Mm00517341_m1), *Mmp20* (Matrix Metallopeptidase-20, Mm00600244_m1), *Amelx* (Amelogenin X-linked, Mm00711642_m1) and *Enam* (Enamelin, Mm00516922_m1). Three independent experiments were performed and the results were essentially identical.

### Generation of WDR72 expression vectors

All vector construction was performed by polymerase chain reaction (PCR) using HotStarTaq DNA polymerase (Qiagen) and site-directed mutagenesis with the megaprimer PCR procedure as we previously reported^45^. The plasmids containing the full length sequence of human WDR72 was purchased from Open Biosystems and used as a PCR template. The sequences of the primers were as follows: forward primer/F1, 5′-GACAATTGATGAGGACTTCCCTGCAG-3′ and reverse primer/R1, 5′-GCCTCGAGTTAAGACACCTTGCAGGGGC-3′. The coding region of wild type (WT) WDR72 with stop codon was amplified by PCR (forward-reverse primers; F1–R1) and the amplified fragment was subcloned into pcDNA3-5′-Flag-tag (pc3-Flag) mammalian expression vector. The plasmid harboring pc3-Flag-WDR72 WT was then obtained. To generate an AI form of WDR72 with c.2934G>A mutation^11^ in the expression vector, which resulted in the nonsense mutation at tryptophan residue 978 (p.W978X), the reverse primer with this mutation (5′-GCCTCGAGTCAACAGGAAATTAGCTTC-3′; R2) was designed. The PCR fragment of WDR72 W978X was amplified by the primers (forward-reverse primers; F1–R2), and the resulting fragment was subcloned into pc3-Flag. The plasmid harboring pc3-Flag-WDR72 W978X was then obtained. To generate CAAX mutation form of WDR72 in the expression vector, which resulted in the missense mutation at cysteine residue 1099 with serine residue (p.C1099S, WDR72 CS), the reverse primer with this mutation (5′-GCCTCGAGTTAAGACACCTTGGAGGGGC-3′; R3) was designed. The PCR fragment of WDR72 CS was amplified by the primers (forward-reverse primers; F1–R3), and the resulted fragment was subcloned into pc3-Flag. The plasmid harboring pc3-Flag-WDR72 CS was then obtained.

All plasmids generated were sequenced, and their DNA sequences were 100% identical to the respective reference DNA sequences available at NCBI or the respective mutations.

### WDR72 protein expression and transfection

To investigate the expression levels of WDR72 protein in ameloblastic cells, ALC and LS8 cells were cultured, cell lysates were extracted using the lysis buffer containing 150 mM NaCl, 20 mM Tris–HCl, pH 7.5, 10 mM EDTA, 1% Triton X-100, 1% sodium deoxycholate and a cocktail of protease inhibitor and prepared in the same manner as previously described^44^. As a control of non-odontogenic cell line, cell lysates from NIH3T3 were also prepared. The HEK 293 cells were plated onto 6 well culture plates at a density of 3 × 10^5^ cells/well and transfected with pc3-Flag-WDR72-WT or an empty vector (pcDNA3-Flag) using X-tremeGENE 9 DNA transfection reagent. After 24 h, cells were lysed and used as controls for the following experiment.

Fifty µg from each cell lysate was incubated with SDS sample buffer and then boiled at 98 °C. The proteins were then separated by 4–12% Bis–Tris Plus SDS-PAGE gel (Thermo Fisher) and electrophoresed. The gel was then transferred to a polyvinylidene fluoride membrane (PVDF) (Immobilon-P; Millipore). Western blot analysis was performed in the same manner as previously described^45^ to investigate the level of WDR72 protein using anti-WDR72 antibody. Chemiluminescent detection of bound antibodies was determined using the ECL Western blotting detection reagents (Amersham ECL Prime, GE Healthcare Life Sciences) and images were acquired using ImageQuant LAS 4000 Biomolecular Imager (GE Healthcare Life Sciences). The membrane was stripped and then re-probed using anti-Flag antibody to detect the presence of Flag-WDR72 WT or anti-β-actin antibody for a loading control.

### WDR72 expression at the cytosolic and membranous fractions in ALC and LS8 cells

ALC and LS8 cells were cultured onto 6 cm dishes and once cells reached confluency, the plates were placed on ice in preparation for cell fractionation. The media were removed and each plate was rinsed with 1 X phosphate-buffered saline (PBS). The cells were mixed with 1 ml of PBS and protease inhibitors (0.83 mM benzamidine, 0.23 mM PMSF, 0.5 µg/ml aprotinin, and 0.5 µg/ml leupeptin), and then collected using a cell scraper. Five hundred µl was kept as the total cell homogenate. To prepare the cytosolic fraction, the other 500 µl of collected cells were cracked by passing through a ball bearing cell cracker 10 times, then transferred to an ultracentrifuge tube and centrifugated for 20 min at 15,000×*g*. The supernatant was then collected and kept as the cytosolic fraction. The remaining cell pellet was resuspended in 500 µl of PBS with the protease inhibitors and kept as the membranous fraction. Finally, 50 µl of Triton-X100 was added to each fraction (total cell homogenate, cytosolic and membranous fractions) and placed on a shaker for 10 min at 4 °C. The fractions were then centrifugated at 10,000×*g* at 4 °C and they were kept at -20 °C until use. Protein concentration was measured using a Detergent Compatible (DC) protein assay kit (Bio-Rad) and a plate reader. Fifty µg of proteins from each fraction were mixed with SDS sample buffer, applied to 4–12% SDS-PAGE, and subjected to Western blot analysis with anti-WDR72 antibody in the same manner as described. The membrane was further stripped and re-probed with anti-Calnexin antibody. Another set of 50 µg of protein samples from each fraction were also prepared, mixed with SDS sample buffer, applied to 4–12% SDS-PAGE, and stained with Coomassie Brilliant Blue R-250 (CBB) (Bio-Rad). The band intensities of WDR72 protein immunoreactive to anti-WDR72 antibody in the cytosolic and membranous fractions of ALC and LS8 cells from three different experiments were measured by the Image J software (NIH). The value of the intensity in the membranous fraction was divided by that in the cytosolic fraction and the % ratio values were calculated, and the mean values ± SD are shown. The asterisks indicate statistically significant difference of WDR72 protein expression between the cytosolic and membranous fractions (p < 0.01).

### Immunofluorescence imaging

For intracellular localization studies, COS-7 cells were plated at a density of 4 × 10^5^ cells on 12 mm poly-l-lysine (PLL)-coated coverslips, no 1.5 (NeuVitro Corporation, Vancouver, WA) on the day before the transfection and transfected using jetPRIME according to the manufacturer’s recommendations. Five hours after addition of the transfection mix to the cells, the medium was removed and replaced with regular culture medium. On the following day, cells were washed with PBS, fixed with 2% paraformaldehyde in PBS for 10 min at room temperature (RT), permeabilized with 0.2% Triton X-100 in PBS for 1 min at RT, and washed with PBS. The primary antibodies (anti-Flag M2, dilution 1:50,000; anti-TGN46, dilution 1:250; anti-AP-1, dilution 1:200) were diluted in 0.02% Triton X-100 in PBS and incubated for 1 h at RT. Cells were washed with 0.02% Triton X-100 in PBS and incubated for 45 min at RT with secondary antibodies diluted in 0.02% Triton X-100 in PBS (goat anti-rabbit IgG-Alexa Fluor555 and goat anti-mouse IgG-Alexa Fluor647, dilution 1:1,000, Thermo Fisher Scientific). After two washes with 0.02% Triton X-100 in PBS and one additional wash in PBS, coverslips were rinsed in distilled water and mounted using Prolong Diamond Antifade (Thermo Fisher Scientific). In some cases, cells were treated with 10 µg/ml brefeldin A for 10 min or 33 μM nocodazole for 3 h prior to fixation. Wide-field fluorescence images were acquired on a Zeiss D1 inverted microscope equipped with Colibri.2 LED and HXP 200 light sources (Zeiss) and an Orca Flash 4.0 camera (Hamamatsu) using the appropriate wavelengths and filter settings.

To determine Golgi-enrichment of WDR72, for each experiment, ~ fifteen cells expressing either WDR72 wild-type or WDR72 W978X deletion mutant were randomly selected. Immunofluorescence imaging was used to determine the distribution of these WDR72 forms within cells and co-staining for the endogenously expressed trans-Golgi marker TGN46 was used to score recruitment of the WDR72 forms to the Golgi. The number of cells with a pool of WDR72 at the Golgi and the total number of cells analyzed for each condition are shown for three independent repeats of the experiment (Set 1–3 in Fig. 5C).

### Cytosolic/membranous fractionation

For biochemical analysis of WDR72 distribution between cytosolic and membranous fractions, HEK293 cells were plated at a density of 1 × 10^6^ cells on 10 cm dishes on the day before the transfection and transfected with 15 μg of plasmid DNA per plate using Lipofectamine 2000 following the manufacturer’s recommendations. Five hours after addition of the transfection mix to the cells, the medium was removed and replaced with regular culture medium. On the following day, cells were washed with PBS, placed on ice and scraped off the plate in 1 ml per plate of PBS supplemented with the protease inhibitors. Cells were lysed by mechanical force using a ball-bearing cell cracker. One aliquot of 500 μl of cell suspension (total cell homogenate) was kept on ice, a second aliquot of 500 μl was spun for 20 min at 200,000×*g* to pellet the membranous fraction. The supernatant (cytosolic fraction) was transferred to a new tube and the pellet was resuspended on 500 μl of PBS with the protease inhibitors. Triton X-100 was added to all fractions at a final concentration of 1% and the samples were rocked for 10 min at 4 °C to allow for membrane solubilization. The samples were then spun for 2 min at maximum speed to remove debris and 100 µl aliquots of the resulting supernatant for all fractions were resolved by SDS-PAGE. Western blot analysis determined the levels and distribution of protein within the total homogenate, cytosolic and membranous fractions. Antibodies used in this study were as follows; anti-clathrin (clathrin heavy chain, dilution 1:500), anti-Akt (dilution 1:1,000), and anti-GM130 (dilution 1:500) antibodies.

### Statistical analysis

Student T-test was used to assess the statistical difference between groups (ALC and LS8) in the gene marker expression analysis (*p < 0.01).

## Supplementary Information

Supplementary Figure S1.
