# Supplementary material for: Identification of the C-terminal region in Amelogenesis Imperfecta causative protein WDR72 required for Golgi localization
Source: Sci Rep. 2022 Mar 17;12:4640. doi: 10.1038/s41598-022-08719-7 (PMC8930991; doi:10.1038/s41598-022-08719-7)
Supplement: Supplementary file 1 — Supplementary Figure S1. [file 41598_2022_8719_MOESM1_ESM.pdf]

Supplementary Information

Identification of the C-terminal region in Amelogenesis Imperfecta causative protein WDR72 required for Golgi localization

Dina Husein, Ahmed Alamoudi, Yoshio Ohyama, Hanna Mochida, Brigitte Ritter, and Yoshiyuki Mochida

Supplementary Fig. S1. Husein et al.

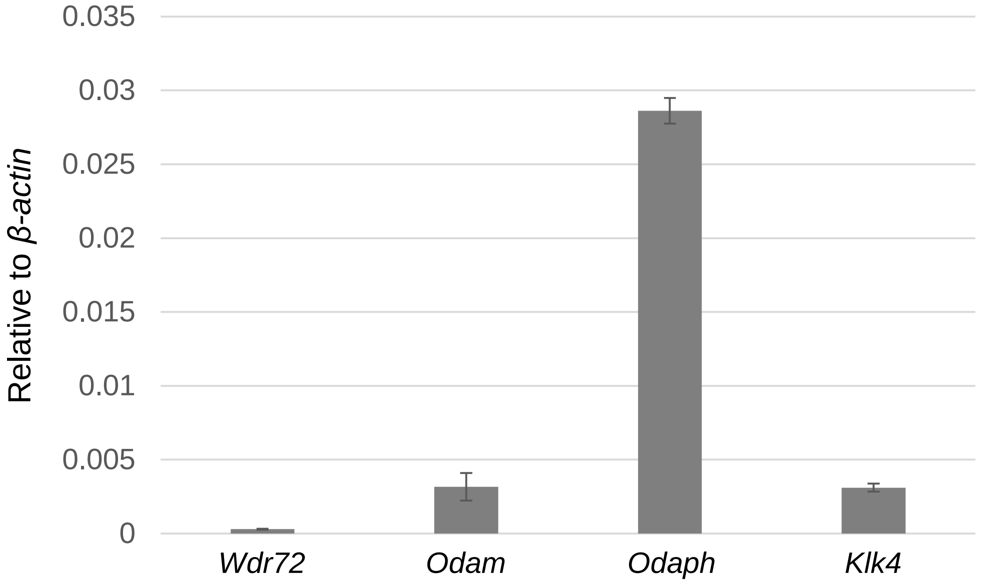

Supplementary Figure S1. Comparison of the amelogenesis gene markers expression in the maturation stage ALC cells.
The expression levels of the amelogenesis gene markers at maturation stage (*Wdr72*, *Odam*, *Odaph* and *Klk4*) were measured by real-time PCR analysis. The expression of each gene (*Wdr72*, *Odam*, *Odaph* and *Klk4*) was compared based on the normalization to that of *β-actin*. The values are shown as the mean ± SD based on the averages of three independent experiments with triplicates in each. *Odaph* expression was found to be the highest followed by *Odam* and *Klk4*, and *Wdr72* was found to be the lowest.

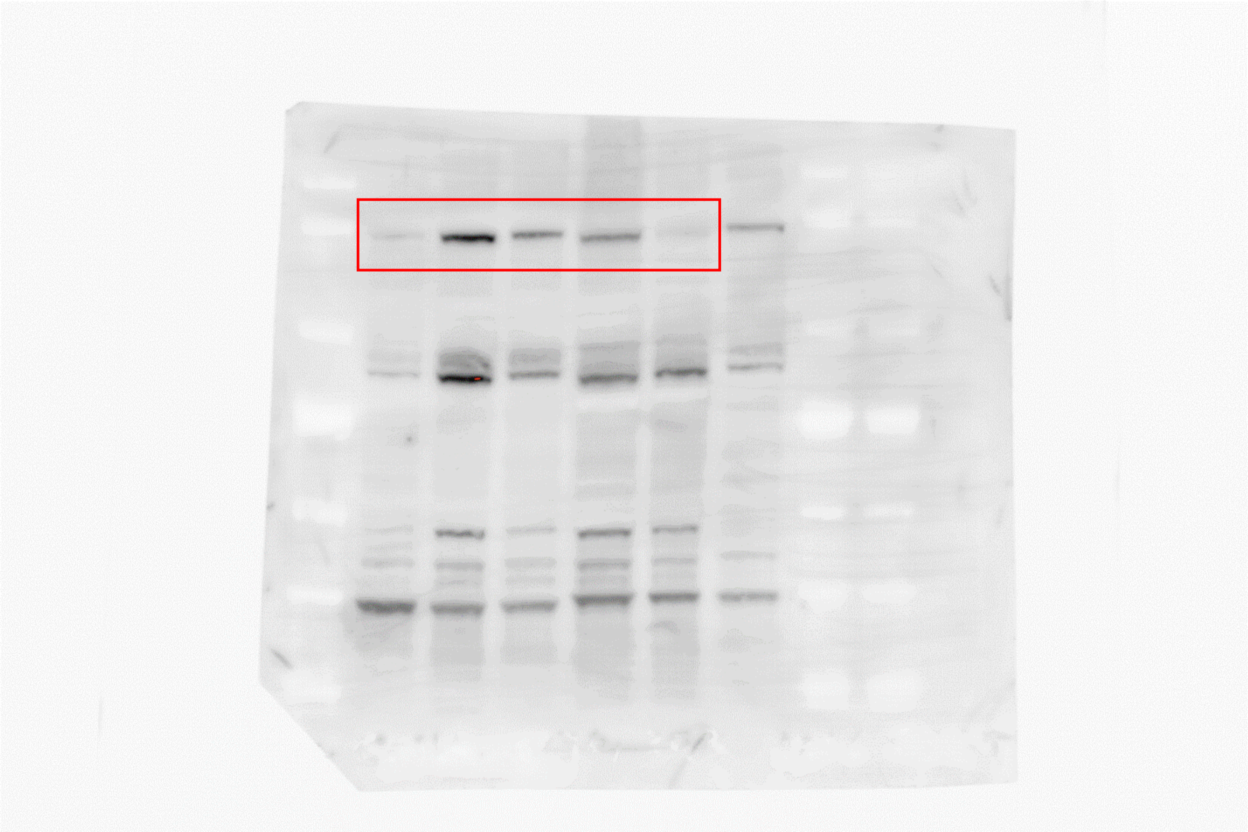

The original blot in Fig. 2B, Upper panel

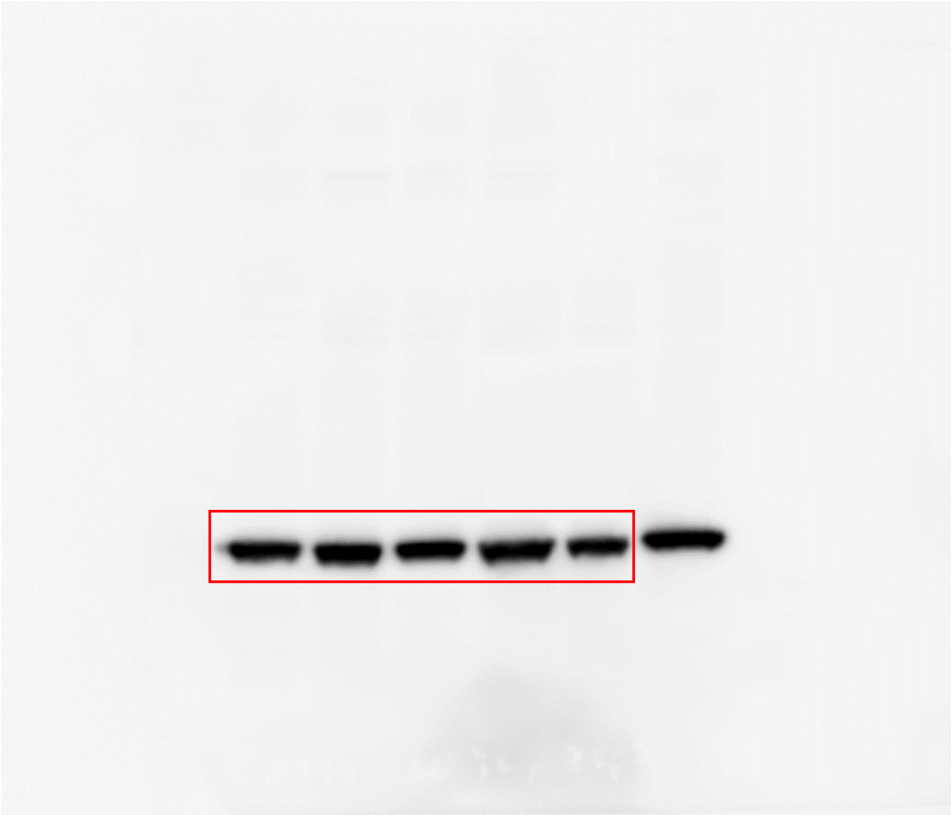

The original blot in Fig. 2B, Lower panel

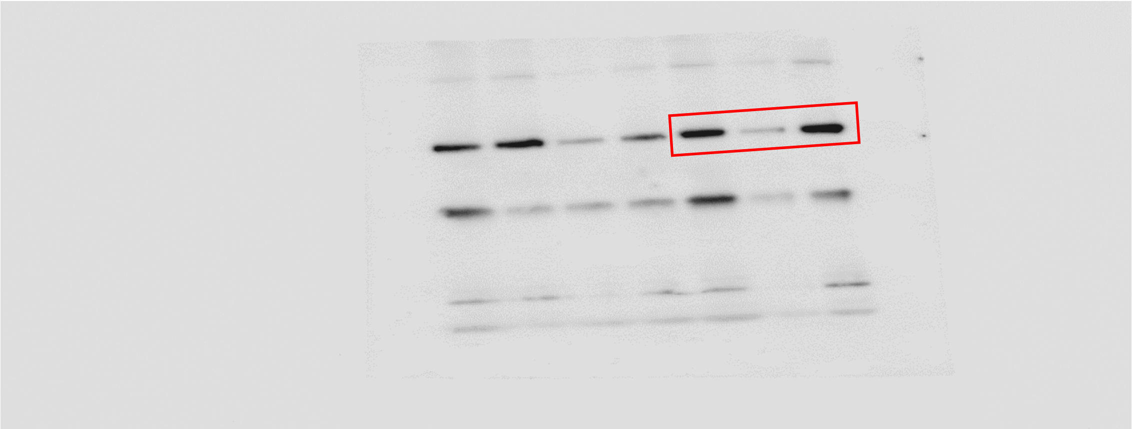

The original blot in Fig. 3A, Upper left panel

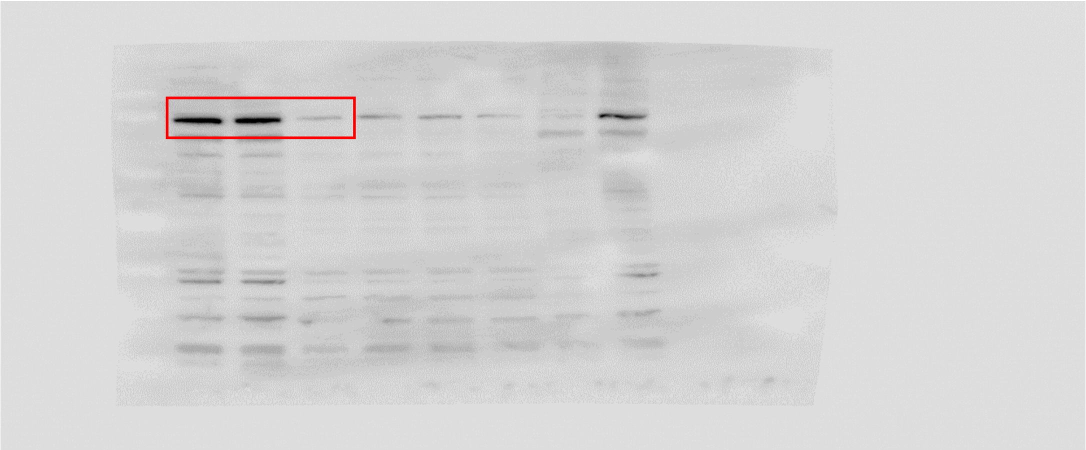

The original blot in Fig. 3A, Upper right panel

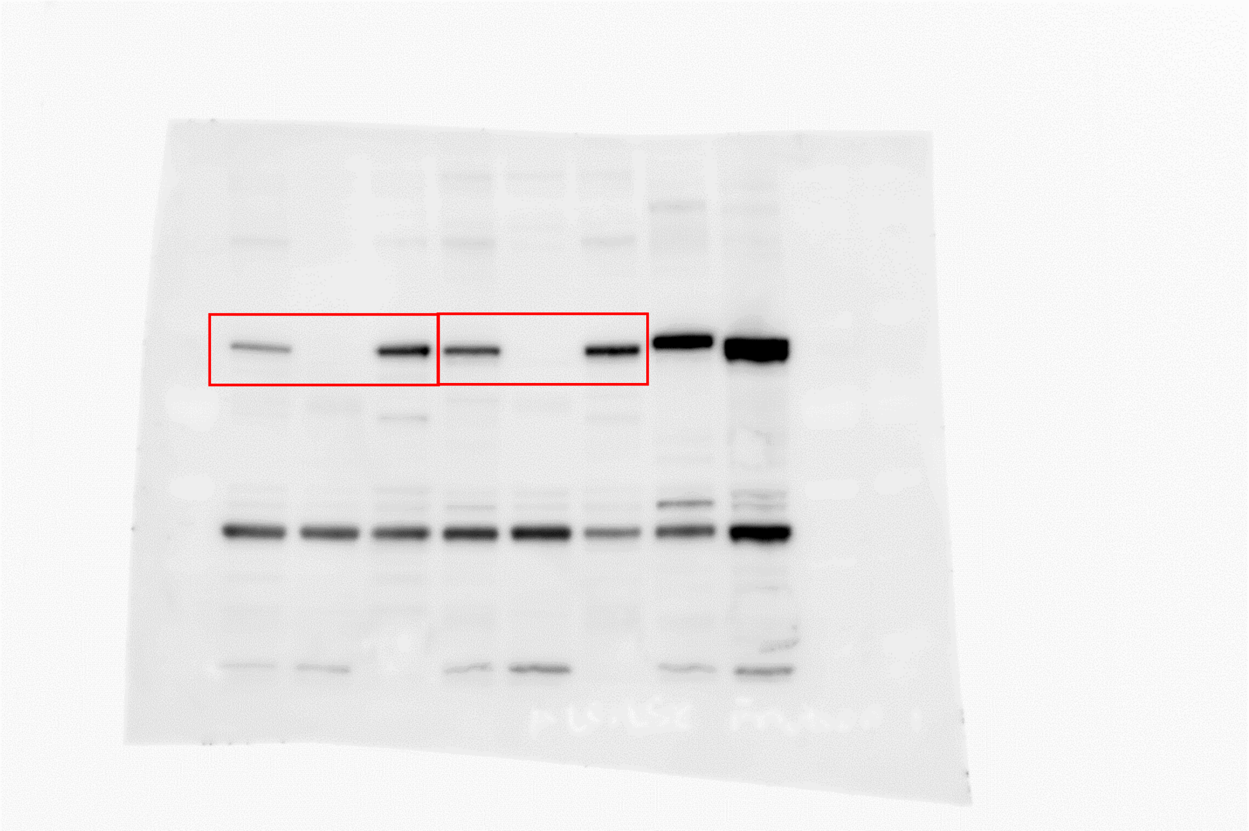

The original blot in Fig. 3A, Lower left and right panels

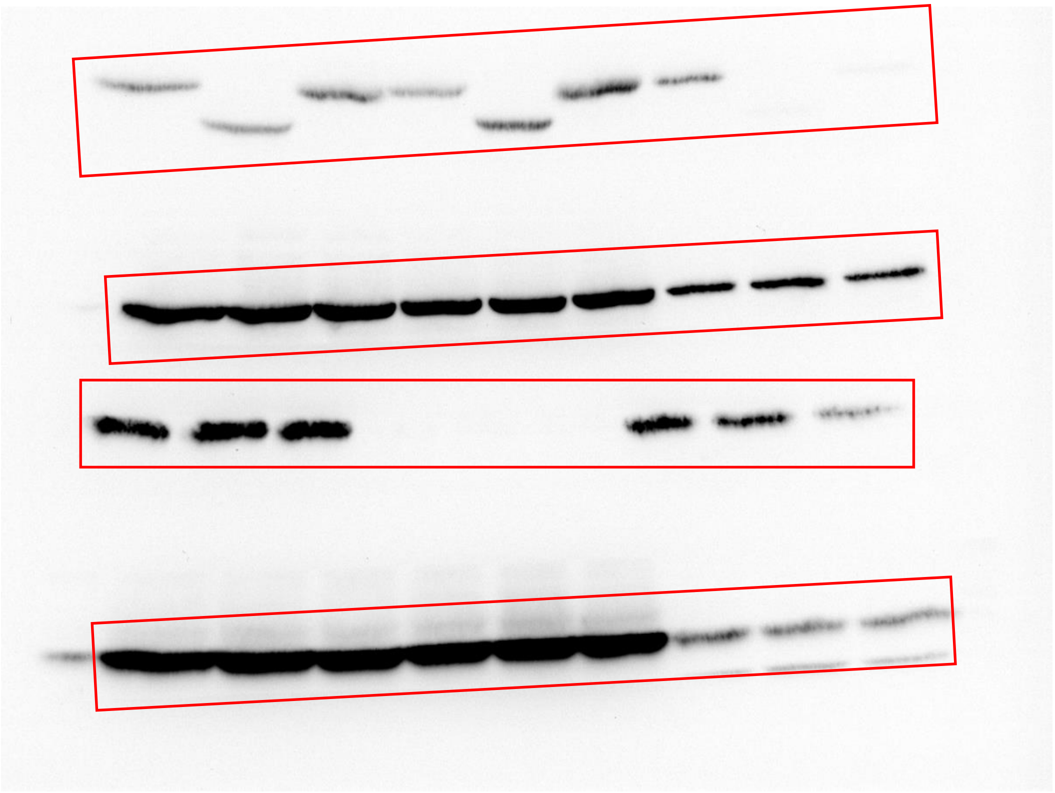

The original blot in Fig. 6B.

Please note:

Top: Flag-WDR72

Middle upper: Clathrin

Middle lower: GM130

Bottom: Akt
